# Book Review

**Published:** 1937

**Authors:** 


### Reviews of Books

**Historical Notes on Psychiatry.** By J. R. Whitwell, M.B. Pp. xii., 252. London: H. K. Lewis & Co. 1936. Price 10s. 6d.—Books dealing with the history of psychiatry are few. We appreciate the issue of this volume. As honorary librarian of the Royal Medico-Psychological Association, Dr. Whitwell has had ample opportunity of gleaning information of old practices. He shows that he has used his good fortune to the full. Passing through early psychiatry in the East and Egypt, he brings us to the Bible, the Talmud and Al Qurân. Next, he gives cases from the classics. The author deals with Pythagoras, Plato, Aristotle and with the classical medical writers to the end of the period he reviews. The period of the Middle Ages receives special consideration, as do also epilepsy, incubus (nightmare) and acedia (manic-depressive psychosis, schizophrenia and anxiety neurosis). The author completes the book with one hundred pages of translations and chronological data. The book is illuminating and fascinating. Many of the hypotheses held to-day date back to the Ancients. As the book terminates at the close of the sixteenth century, the complete growth of psychiatry is not described. We hope that a companion volume will be issued bringing the subject up to the present time. The book is published with the usual efficiency of Messrs. H. K. Lewis & Co.'s publications. Unfortunately, there is no index.

**The Natural History of Disease.** By John A. Ryle, M.D., F.R.C.P. Pp. x., 438. London: Oxford University Press (Humphrey Milford). 1936. Price 15s.—Professor Ryle, who is now Regius Professor of Physic in Cambridge, has collected into a single volume many addresses and articles which he has delivered and written during the past ten or fifteen years. They illustrate admirably the genius for critical observation at the bedside which is the mint-mark of Guy's Hospital. It is not so much the discovery of new symptoms or new diseases that one learns from Ryle's writing, it is the formed habit of first-hand observation that is worth studying and, according to the limits of one's ability, that is worth copying. In one of his addresses (on research in clinical medicine) he speaks of the great discoveries made by medical men who were "observational" rather than "experimental" in their methods of research. These essays are illustrations of the "observational" method written as though they are intended for leisurely reading. They are full of sound practical hints, the outcome of wide and varied experience which the general practitioner as well as the physician will enjoy and find profitable.

**Elementary Genetics.** By H. GRÜNEBERG. Pp. iii., 87. Illustrated. Edinburgh: E. & S. Livingstone. 1937. Price 1s. 6d.—The appearance of this little book in the Catechism Series, and its full title *Elementary Genetics for Students of Biology and Medicine*, will, it is to be feared, deceive many purchasers. It is accurate, remarkably complete, thoroughly up to date and for the most part very clearly written, but it is highly condensed, and it is certainly not elementary. The student of biology who has taken a comprehensive course in genetics will find it most valuable for revision and, small though it is, it need not be despised by the student who is about to take an honours examination in botany or zoology. To the medical student its appeal is more dubious. It is possible that the biology course at some medical schools might include as much genetics as is covered here, but that is most unlikely to be the case at the majority. The medical student in his final years who wishes to revise the subject in order to understand the rôle of heredity in the production of abnormalities and diseases will, it is to be feared, find the book much too difficult.

**The Elements of Medical Treatment.**—Third Edition. By R. HUTCHISON, M.D., LL.D., F.R.C.P. Pp. viii., 194. Bristol: John Wright & Sons, Ltd. 1937. Price 5s.—The third edition of this favourite book has been brought up to date with some minor alterations and additions. The chief alteration lies in the chapter on diabetes, which has been rewritten and constitutes a great improvement on the second edition. It is a pity that the book went to press before the introduction of protamine-insulin, so that this valuable improvement in insulin treatment is not mentioned. Of the other additions the more important are the inclusion of a paragraph dealing with dyschezia as a cause of constipation, a description of the use of mandelic acid as a urinary antiseptic, and several references to Salyrgan and its allies. It is disappointing that so important a method of oxygen-administration as the "oxygen tent" has escaped notice.

**High Blood Pressure.** By I. HARRIS, M.D. Pp. xx., 132. London: Oxford University Press (Humphrey Milford). 1937. Price 10s. 6d.—The problem of the ætiology of essential hypertension has been examined by Dr. Harris and his team of workers by an elaborate series of investigations on the arterial pressure and the bio-chemistry of the blood and urine of a group of about twenty-five patients. In particular alterations in these were investigated under conditions of variation in the protein content of the diets. The net outcome of these researches is the conclusion that the essential cause of most cases of hyper-tension is to be found in excessive protein intake. In the development of this thesis Dr. Harris is led to much interesting theorizing and occasionally to some rather startling assertions. The greater part of the book is taken up with detailed discussion of the extensively tabulated results of experiments. These do not lend themselves to light reading, and the work is obviously for the close study and concentrated attention of the serious research student working in the fields of arterial pressure and kidney diseases.

**Diagnosis and Treatment of Venereal Diseases.** By DAVID LEES, D.S.O., F.R.C.S., F.R.C.P., F.R.S.E., edited by ROBERT LEES, M.B., F.R.C.P. Third Edition. Pp. xvi., 608. Illustrated. Edinburgh: E. & S. Livingstone. 1937. Price 15s.—Of all the diseases which occasionally meet with comparatively inefficient treatment venereal diseases probably take first place. Lack of knowledge would be no excuse after perusal of this work. Perhaps a more apt title would be "Diagnosis and Treatment of Venereal Diseases in the Male," for the space devoted to these conditions in regard to the female is relatively small and not as complete as is found in many works on gynæcology, while that part devoted to the venereal diseases in the male is comprehensive, though as brief as efficient description will allow. The book is written in a manner which is sure to make the subject clear to the most elementary student, a style not too common in medical literature. A chapter on the technique of the collection of specimens is particularly helpful. Many of the illustrations are excellent, but there are some which might be omitted or with advantage replaced by something more enlightening ; for instance, there are two excellent photographs of a doctor with his eye to the eyepiece of a urethroscope : much more information would be imparted if the reader had a visual impression of what he, the doctor, saw at the other end. Here and there are statements which could not meet with universal approval in the profession, but such things are stimulating to thought and argument, and are not therefore to be deplored. There is much useful information in its pages, and the book should become popular among those for whom it is written.

**Common Skin Diseases.** Fourth Edition. By A. C. ROXBURGH, M.D., F.R.C.P. Pp. xxxii., 401. Illustrated. London: H. K. Lewis & Co., Ltd. 1937. Price 15s.—Roxburgh's *Common Skin Diseases* now holds such an established position among text-books of dermatology that little need be added to what has already been said in previous reviews. The production of a Fourth Edition within five years of the original publication speaks for itself. While not pretending to give an account of every skin disease it provides the student and practitioner with the essential details of diagnosis and treatment of those diseases which he is most likely to meet either in practice or in the examination room. The new edition contains twenty-six additional pictures, while the sections on erythema nodosum, urticaria and eczema have been modified in accordance with the most recent views on these subjects. The sections on varicose veins and skin conditions resulting therefrom has been revised by Mr. R. T. Payne, who has added a short account of the contra-indications to injection treatment. There can be little doubt that this is quite one of the best, if not the best, of the smaller books on the subject, which will amply repay the money spent on it.

**Diagnosis of some Delusional Insanity Types in General Practice.** By E. HOPEWELL-ASH, M.D. Pp. 64. London: John Bale, Sons & Danielsson. 1936. Price 2s. 6d.—The sub-titles of this booklet are somewhat confusing until one finds that the author reserves the term dementia præcox for a type of schizophrenia. The manic-depressive states are also considered. The plan of giving differentiating tables is useful, but the tables are not always accurate. For instance, depression and elation in manic-depressive states are not always logically explained. Depression may descend suddenly " like a blanket " for no apparent reason. The author gives good case descriptions, but his use of repetition is somewhat tiresome. The booklet is written with the idea of assisting the general practitioner, and should, on the whole, prove useful. Unfortunately, although it is of pocket size, the paper cover and weak binding prevent it from being carried frequently.

**The Common Neuroses.** By T. A. Ross, M.D., F.R.C.P. Second Edition. Pp. xii., 236. London: Edward Arnold & Co. 1937. Price 10s. 6d.—A second edition of this practical book is overdue, and we welcome this more mature volume. Few books are its equal for common-sense information. In places the author appears to be using on his readers methods perhaps applicable to patients. For instance, doubt might be cast upon the accuracy of the statement that everyone has discomfort nearly every day. The statement may be true of those who are constitutionally manic-depressives when in their melancholic phases, but not to the majority of mankind. Trotter's remark that severe pain occurs to almost everyone every day is quoted as if unquestioned. However, the flaws are few, and the book will be useful to those practising the more elementary psychotherapy, but the treatment described could be undertaken by few general practitioners. The majority would be unable to give at least an hour to one patient without fear of disturbance by the telephone. The point is omitted by the author, yet a disturbance may interfere inconveniently with the process of free association. On the whole the book is well produced, and the index is valuable.

**Diseases of the Testicle.** By HAMILTON BAILEY, F.R.C.S. Pp. viii., 160. Illustrated. London: H. K. Lewis & Co. Ltd. 1936. Price 12s. 6d.—This small monograph is well up to the very high standard which has been set in the past by Mr. Hamilton Bailey's previous works. It is comprehensive and covers almost every aspect of the subject, and at the end of each chapter there is an admirable bibliography. It is excellently illustrated; the printing is well done and the indexing is accurate and detailed. As an easy and interesting way of covering the whole of this subject in a short time it is outstandingly good, and can be read without difficulty in a couple of evenings. The author and publishers are to be congratulated on the production of this handy little book.

**Hay Fever.** By CLIVE SHIELDS, B.M., B.Ch. Pp. viii., 58. Illustrated. London: Oxford University Press (Humphrey Milford). 1937. Price 7s. 6d.—This book is devoted to the advocacy of zinc ionization for the relief of hay fever, by the author's method. This method has in some mysterious way become rather a favourite in the lay press, possibly because of the anonymous book published for laymen on the same subject and at the same time. The treatment itself is by no means new—in the nineties it was described as going out of favour. But since many patients ask for it, a description of the technique has a definite value. The author appears to believe that this method is the only one worth considering, and to have a measure of success that his imitators must envy. But is it necessary to include a table to show that none of his patients have been relieved before coming to him? There are four photographic plates, a sketch of the innervation of the nose, too diagrammatic to be useful; and a cross section of the nose of that curious creature, the mugwump, adds to the interest of page 11. Four and a half pages of index seems an excessive allowance for 51 pages of text.

**Obstetric Technique.** By F. J. BROWNE, M.D., Ch.B., D.Sc., F.R.C.S., F.C.O.G. Pp. ii., 70. Shrewsbury: Wilding & Son Ltd. 1937. Price 2s.—This hand-book deals in detail with the technique to be observed by nurses and students in the Obstetric Department and district of the University College Hospital, London. The instructions are clear and well thought out, and are a model for any obstetric department. The management of labour and the treatment of abnormalities of pregnancy and labour are concisely defined. It is a very useful hand-book for obstetric clerks and nurses, but is not of universal application owing to the varying methods in use in different hospitals.

**Safe Childbirth.** By K. O. VAUGHAN. Pp. x., 154. Illustrated. London: Messrs. Baillière, Tindall & Cox. 1937. Price 7s. 6d.—This book, which makes interesting reading to all engaged in active midwifery, shows evidence of much research and thought, and will no doubt stimulate further interest in the bony pelvis, particularly in our conception of its shape and mobility. Dr. Vaughan claims that the brim of the normal pelvis is round, not heart-shaped. She certainly gives us fresh thoughts on this subject, and her views, which are illustrated by numerous diagrams and photographs, are well supported by much data and information, gained by personal investigation of the customs of primitive peoples and numerous nations with their different classes and modes of living. Whether we agree with her conclusions or not, they certainly demand attention. In speaking of the puerperium, Dr. Vaughan is less certain of her facts, as most patients nowadays do active exercises during the puerperium and rarely lie flat on their backs except for very short periods, being nursed mainly in the Fowler position.

**The Medical Cookery Book.** Compiled by DOROTHY SEWART. Pp. 136. Bristol: John Wright & Sons Ltd. 1935. Price 2s. 6d.—This book contains 300 recipes "For the use of invalids, convalescents and children," and should be of great use to those responsible for their feeding. Economy and common sense are evident, and the methods described are on the whole simple. The book is practical and full of ideas, and one can imagine a most interesting menu such as liver cocktail, peptonized oysters, raw egg, *served without comment*, and a porter jelly.

**Milk Products.** By W. C. HARVEY, M.D., D.P.H., M.R.San.I., and H. HILL, M.R.San.I., A.M.I.S.E., M.S.I.A. Pp. viii., 387. Illustrated. London: H. K. Lewis & Co. Ltd. 1937. Price 16s.—The subject of milk has furnished quite a number of writers with a popular topic, and many authoritative books covering practically every aspect of this most essential food are available; but the products of which milk forms the prime basis and which, as a consequence, are equally important, appear hitherto to have been neglected. The authors have recognized this defect, and having themselves recently contributed a valuable text-book on the subject of milk, felt compelled to supply the want: and this book makes a welcome appearance as a companion treatise to their own *Milk-Production and Control.* The place of importance in the first chapter is devoted to ice-cream. It is rightly given prominence and most efficiently dealt with. Commencing with an historical survey, its food value, sources of bacteria, the scientific investigation of epidemics and diseases transmitted by ice-cream are all exhaustively dealt with. The method of manufacture is fully explained, and the analytical and bacteriological data are valuable sections. Practical suggestions are made for more efficient legislative control. The other principal products of milk are dealt with in separate chapters, each compiled on the same standard plan, so that adequate comparison can be made from section to section. The book is well written and the matter presented in an interesting and easily readable form. It is well interspersed with illustrated diagrams and completed with a very full index. *Milk Products* is essentially a public health text-book, and will prove of special value to Medical Officers of Health, Sanitary Inspectors and Public Health officials generally, whose duties are concerned with the supervision of our food supply.

**The Diet Book for Doctor, Patient and Housewife.** By M. R. REA. Third Edition. Pp. xvi., 255. London: Oxford University Press (Humphrey Milford). 1937. Price 6s.—Although the first edition of this book appeared in 1931 this is the first edition that has been sent to us for review. In his foreword to the first edition Sir James Purves Stewart described it as an ingenious and carefully thought out diet book. This is certainly a true description. Mrs. Rea has evidently wide practical experience and realizes that invalid cookery has suffered much in the past from want of variety. The reader's first impression may be that there are many items that are unknown to British cookery, that many of the dishes sound too foreign for English palates. But when one reflects on the insipidity and sameness of the invalid diets which are customary in this country, when as doctors we recall the difficulties of persuading patients to endure the dietetic restrictions we are compelled to order, Mrs. Rea's skill in arranging varied menus for invalids will be warmly appreciated. Not the least valuable part of her book consists in her wide range of cookery recipes which do not entail either too expensive or too elaborate preparations. Here and there she omits to explain how a particular article is prepared, as, for example, when she ends the recipe for the delicious soup known as "Bortsch" with the remark "Pirozhkis are a good accompaniment." Careful search through the book and its index has failed to reveal what "Pirozhkis" are. Another minor criticism suggests itself in the Obesity Dietary; it is assumed that the patient has the discretion to know how much he shall eat, but there is no hint of any limit to the amount of lean meat, fish and dry cheese that is advisable. Moreover it is stated that Vita Weat and Ryvita may be taken with all meals, no quantities being given. Yet there are general directions that starchy foods should be restricted. Now the carbohydrate content of Vita

Weat and Ryvita is in each case 21·2 grammes per ounce, whereas the carbohydrate content of white bread is only 15·5 grammes per ounce. It may be that white bread is more tempting to the palate and more of it is likely to be eaten, but no such explanation is given to the patient or the doctor. Again, there is a special warning against potatoes, but boiled potatoes either new or old contain less than 6 grammes of carbohydrate per ounce. These oversights are probably not Mrs. Rea's but those of Marcovici, whose dietaries she quotes. As an invalid cookery book this is to be commended most highly.

**Modern Dietary Treatment.** By MARGERY ABRAHAMS, M.Sc., and ELSIE M. WIDDOWSON, B.Sc., Ph.D. Pp. ix., 328. Baillière, Tindall and Cox. 1937. Price 8s. 6d. net.—Here at last is a book of dietetics based on English foods and English cookery! That does not mean that our foods or our ways of cooking are the best; what it means is that we are no longer dependent on Sherman's tables, which, admirable as they are, are not accurate analyses of English foods as our patients eat them. The food tables in this book have been compiled from the analyses made by Dr. McCance and his co-workers under the auspices of the Medical Research Council. In many instances the values differ so widely from those given by other authorities as to render these new tables indispensable to all doctors and dietitians in the British Isles. The general principles of nutrition are briefly and clearly laid down in the opening chapters. There follow chapters dealing with diets appropriate to certain diseases. More than half the book consists of detailed diets with cooking recipes. The variety allowed is excellent, all the dishes are those which are customary in this country. The contents of diets in protein, carbohydrate and fat are, where necessary, clearly calculated. There are many valuable tables at the end of the book. Unhesitatingly we place this work as being the most scientific of the smaller dietetic handbooks published in this country; and this is not altogether unexpected, seeing that the authors are dietitian to St. Bartholomew's and bio-chemist to King's College Hospitals respectively.

